# Long-Term Changes in the Abundance, Size, and Morphotype of Marine Plastics in the North Pacific

**DOI:** 10.1021/acs.est.4c09706

**Published:** 2025-02-26

**Authors:** Kentaro Miyazono, Kazuaki Tadokoro, Gajahin G. N. Thushari, Hiroomi Miyamoto, Akinori Takasuka, Mikio Watai, Tohya Yasuda, Takuya Sato, Rei Yamashita, Taketoshi Kodama, Kazutaka Takahashi

**Affiliations:** † Graduate School of Agricultural and Life Sciences, The University of Tokyo, 1-1-1, Yayoi, Bunkyo, Tokyo 113-8657, Japan; ‡ Japan Fisheries Research and Education Agency, 3-27-5 Shinhama-cho, Shiogama, Miyagi 985-0001, Japan; § Department of Animal Science, Faculty of Animal Science and Export Agriculture, Uva Wellassa University, Passara road Badulla, Badulla 90000, Sri Lanka; ∥ Japan Fisheries Research and Education Agency, 2-12-4, Fukuura, Kanazawa-ku, Yokohama, Kanagawa 236-8648, Japan; ⊥ Bioinformatics Center, Institute for Chemical Research, Kyoto University, Gokasho, Uji, Kyoto 611-0011, Japan; # Atmosphere and Ocean Research Institute, The University of Tokyo, 5-1-5, Kashiwa-no-ha, Kashiwa, Chiba 277-8564, Japan

**Keywords:** plastic pollution, microplastic, fragmentation, sedimentation, Pacific decadal oscillation

## Abstract

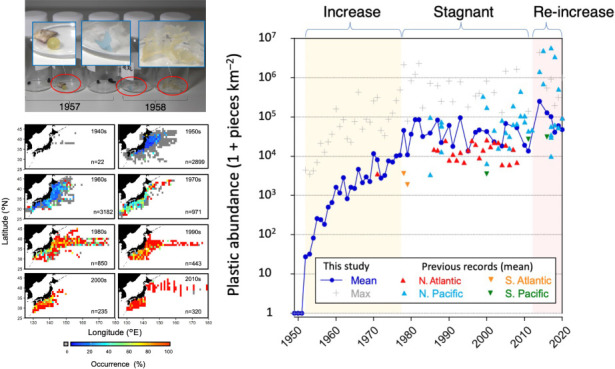

Understanding the spatiotemporal dynamics of microplastics on the ocean surface is crucial for assessing their impact on marine ecosystems and human health; however, long-term fluctuations have not been extensively studied. We present a long-term empirical data set on floating marine plastic debris collected from 1949 to 2020 around Japan in the western North Pacific. We observed three phases: 1) a period of increase (0–10^4^ pieces/km^2^) from the early 1950s to the late 1970s; 2) a stagnation period, with high abundance (10^4^–10^5^ pieces/km^2^), from the 1980s to the early 2010s; and 3) a period of reincrease (>10^5^ pieces/km^2^) from the mid-2010s to the present. The shift from film to fragmented plastic in the 1980s and the continuous downsizing may have caused the expansion of the offshore polluted area, resulting in a stagnation period by enhancing removal. The removal is most likely caused by sedimentation with phytoplankton, as the abundance of the plastic debris during this period was significantly related to the winter Pacific Decadal Oscillation, an index of annual primary productivity. The recent increase in microplastics suggests that plastic discharge is outpacing its removal capacity, suggesting that the impact of pollution on ocean surface biota is becoming increasingly evident.

## Abstract 2

Minimal research exists on the temporal dynamics of microplastics on the ocean surface. This study presents long-term fluctuations in floating plastic debris from 1949 to 2020 in the western North Pacific.

## 1 Introduction

Marine plastic pollution is a serious global environmental issue. This problem is highlighted by the lower-than-expected amount of plastic debris floating on the ocean surface layer (OSL), known as the “missing plastic” phenomenon,^1,2^ which refers to the challenges in tracking plastic waste. This discrepancy could be due to the overestimation of input estimates and underestimation of the significance of plastic removal from the OSL, including fragmentation.^3^ Several studies reassessed plastic discharge from possible sources^4,5^ and removal/fragmentation processes at the OSL.^6−8^ However, the dynamics of the large amounts of plastic that have entered the oceans remain unclear; notably, land-based sources of plastic debris transported by river systems contribute 80% of the total plastic debris in the marine environment.^9^ Microplastics of <5 mm have been detected in close proximity to areas of industrial activity and in remote oceanic regions, being detected in the atmosphere and from the OSL to deep trenches.^10,11^ Notably, plastic contamination of the food chain is a significant concern.^12^ Basic questions about the location of plastic debris and its life cycle in ocean water remain somewhat unanswered; this limits accurate assessment of the impact of pollution on marine ecosystems and human health.

Plastic debris floating on the OSL can enhance our understanding of the dynamics of marine plastic debris, given that once plastics enter the ocean, the OSL water acts as a reservoir for all the buoyant plastic debris and plays an important role in transporting them to remote areas and decomposing them, while dispersing the associated chemical pollutants primarily via wave action, heat, and ultraviolet (UV) radiation, thereby increasing the risk of food-chain contamination.^10,12^ Although the amount of plastic debris on the OSL has increased over time, the long-term trend of marine plastic pollution is unexplored. Archived continuous plankton recorder (CPR) samples from the North Atlantic indicate an increasing trend in macroplastic entanglement since the late 1950s; however, the subsequent increasing pattern is ambiguous, as it is semiquantitative, in terms of the frequency of occurrence.^13^ As the earliest quantitative record was obtained using plankton net sampling in 1972,^14^ the abundance of plastic debris in the OSL seemed to increase from 10^3^ to 10^4^ pieces/km^2^ until the mid-1980s in the North Atlantic^15^ and North Pacific;^16^ however, several studies reported no temporal trend in plastic abundance in the OSL until the 1990s to mid-2000s.^17,18^ Furthermore, plastic abundance in the OSL has increased abruptly since the mid-2000s,^19,20^ suggesting a global trend.^18^ However, interpreting snapshots of plastic abundance on a long-term basis is challenging because of the gaps in the spatiotemporal data for a region, variations in sample collection and station selection, and differences in study techniques and methodologies, which complicate the debris-detection process.^18^ Microplastics in marine environments portray diverse morphotypes, e.g., spheres, fragments, and fibers, generated by deterioration of larger plastic debris. The morphotypes and sizes of plastics determine their buoyancy and are closely linked to their transport and removal from the OSL; therefore, studying the historical changes in these processes can be the key to understanding the evolution of plastic pollution in the marine environment. However, the current literature reveals no relevant information.

Hence, we present the longest empirical data set for the plastic debris collected from the OSL (using plankton nets) from 1949 to 2020, covering the period from the onset of plastic pollution to the current situation around Japan in the western North Pacific (WNP). Notably, our study is the first to analyze the associated temporal changes in the size and morphotype of the plastic debris in the region, especially as these characteristics determine the dynamics of marine plastic pollution (via dispersal and removal/fragmentation).

## 2 Materials and Methods

### 2.1 Sampling and Analysis

We analyzed the surface-towed plankton net-sample series (from 1949 to 2020) collected by 31 organizations and research institutes that participated in different monitoring programs for eggs, larvae, and early juveniles of small pelagic fish in Japan, conducted by the Fisheries Research Agency. We selected a total of 9362 samples collected from 298 cruises in an area between 125° E and 180° E and 24° N and 47° N (Table S1). Among these, 8922 samples collected from the area within 131° E–160° E and 25°–47° N were specifically used for the long-term variation analysis of the abundance, size, and shape of plastic debris around Japan (Figure S1). After the onboard sorting of eggs and larvae of small fish, the remaining samples, including those of plastics, were preserved in a 0.5–2-L glass/plastic jar containing formalin seawater (5%). The series of samples were archived by the Japan Fisheries Research and Education Agency in Shiogama, Miyagi, in a dark and temperature-controlled room until analysis.

The plastic samples were manually sorted from the plankton samples according to the method described by Ogi and Fukumoto;^21^ the majority of the plastic particles collected were less than 10 mm in size, although some were substantially larger. The floating objects on the surface were removed from each sample bottle with a pair of tweezers, a glass pipette, and nylon mesh (200 μm). After removing all recognizable floating plastics, the remaining contents were gently mixed several times to check for the presence of plastics entangled with planktonic organisms. Then, the plastic samples were passed through a nylon mesh (100 μm), washed with distilled water, and then dried on filter paper at room temperature (23–26 °C). After drying, all plastic particles were counted and categorized by size, based on their maximum length (ranging from <1 mm to >30 mm) and shape (fragment, bead, pellet, filament, film, and foam) (Figure S2). As for filamentous plastic, we only counted monofilaments to minimize the overestimation by the contamination of nonsynthetic microfibers like cellulose/cotton.^22^ Moreover, monofilaments with similar characteristics (color, thickness, and surface morphology) to those used in the plankton nets made by nylon mesh were also excluded from the counting, and thus, the contaminations seem to be negligible even if they occurred. Polymer composition was not analyzed as the particles would need to be destroyed by pressing with the probe for Fourier transform-infrared spectroscopy–attenuated total reflectance analysis; we needed to preserve the original plastic particles for further analysis of the surface structure and sessile organisms.

During the monitoring program, three different types of plankton nets were used, depending on the study period (Table S1): 1) a “Maruchi” net^23^ was used to assess the particles discharged during 1949–1988; it consisted of a conical net with a 130 cm diameter (at the mouth), 450 cm in total side length, which consisted of the frontal (300 cm) cotton spliced net (2 mm) and the posterior (150 cm) coated with resin GG54 (330-μm mesh aperture). 2) A “Shin-Chigyo” net^24^ was used to assess the particles discharged during 1987–2000; it consisted of a cylindrical-conical net with a 130 cm diameter (at the mouth), 540 cm in total side length, and 450-μm mesh aperture. 3) A “new-neuston” net,^25^ which was modified from the Manta net^26^ was used to assess the particles discharged during 2000–2020; it consisted of a frame of 130 cm (width) and 75 cm (height), 540 cm of total side length, and 450-μm mesh aperture. The net was towed at the air-sea interface off the port side. Notably, for the Maruchi and Shin-Chigyo nets, the net mouth should come up to a position that is convenient for hoisting the nets up; one-third of the mouth diameter was kept out of the water. When the exposed portion of the net was 40 cm at the greatest height, the mouth area underwater was approximately 1 m^2^ (same as the dimension for the new-neuston net, 0.98 m^2^). The tows were conducted at a ship speed of two knots for 5 min (Maruchi net) and 10 min (Shin-Chigyo and new-neuston nets); the resulting areas per sample were 402, 803, and 870 m^2^, respectively. The net was not equipped with a flowmeter, and the filtering efficiency was assumed to be 100%. Although it is not possible to assess the error of estimation in areas per sampling for past observations, quantitative assessment for the recent monitoring program with a new-neuston net using a flowmeter showed the median value of the swept area was 0.996 and thus closely approximated the theoretical value of 1.0.^27^ No corrections for the effect of wind mixing were considered because of the considerable shift in plastic morphotypes, which could be a possible source of error in detecting historical changes in plastic abundance and composition.

Plastic concentration for each tow was computed as the total number of collected pieces divided by the tow area, reported in units of pieces/km^2^. No calibration was conducted between the Shin-Chigyo and new-neuston nets, as there was no statistical difference in their catch efficiencies.^25^ The data for the Maruchi net were calibrated using a selection curve,^28^ based on the data obtained from the two nets (Maruchi net and Shin-Chigyo net) during 1985–1990. The results indicated that the catch efficiencies for particles smaller than 2.8 mm were consistently lower for the Maruchi net (Figure S3). We applied a correction factor (*C*) for the number of plastics for each size class (*x*) collected by the Maruchi net, using the following equation:
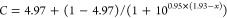


The underestimation of particles smaller than 2.8 mm for the Maruchi net was corrected to the same level as that of the Shin-Chigyo net by applying the equation. Then, we converted the microplastic concentration to a total particle weight concentration (weight per unit area; mg/m^2^), based on the empirical relationships between size (mm) and weight (mg), derived from the samples collected during 1987–2000 for different morphotypes, including granular fragment, flat fragment, film, filamentous, foam, and pellet (Figure S4).

### 2.2 Historical Data Set of Plastic Production and Climate Indices

To examine the possible factors influencing the historical fluctuation of floating plastic debris around Japan, we obtained the annual production data of plastic materials in Japan from 1949 to 2020, compiled by the Japan Plastic Industry Federation (https://www.jpif.gr.jp/english/index.html). Time series data of the total population in Japan from 1949 to 2020 were obtained from the database at the portal site of official statistics of Japan (https://www.stat.go.jp/english/data/jinsui/2.html). The data on global production and use were obtained from the OECD website of Global Plastic Outlook ©OECD 2022 (https://www.oecd-ilibrary.org/environment/global-plastics-outlook_de747aef-en) from “Fig. 2.2 version1” (https://stat.link/r9vlpe). The data of annual variation of days with heavy rain in Japan from 1976 to 2022 (https://www.data.jma.go.jp/cpdinfo/extreme/csv/amdday400mm_p.csv) and that of the Pacific decadal oscillation (PDO) index during winter (December, January, and February) (https://www.data.jma.go.jp/kaiyou/data/shindan/b_1/pdo/pdo.txt) were obtained from the website of the Japan Meteorological Agency (https://www.jma.go.jp/jma/indexe.html) accessed on February 29, 2024. We also obtained the data on long-term changes in microplastics in bottom sediments collected from Beppu Bay, western Japan, from the Supporting Information of Hinata et al.,^29^ as an index of the annual change in microplastics removed by phytoplankton aggregation from surface waters. The relationships between the PDO during winter (mean of December, January, and February) and the logarithmically transformed (log 10(*X* + 1)) abundance of floating plastic debris (*X*: pieces/km^2^) during the stagnant period (1978–2011), which was determined as a period wherein the plastic abundance fluctuated between 10^4^ pieces/km^2^ and 10^5^ pieces/km^2^, were also examined.

## 3 Results

**Figure 1:**
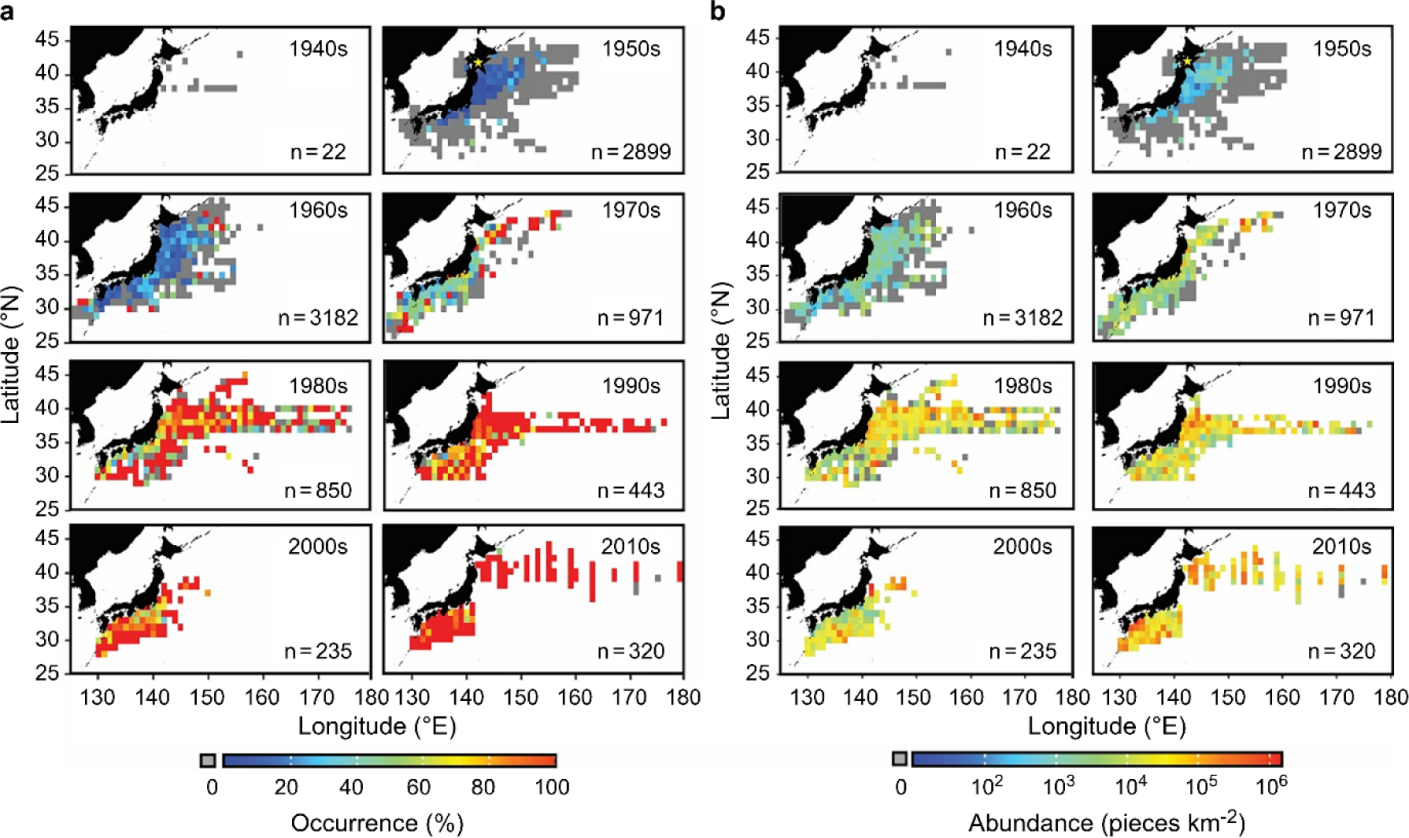
Horizontal evolution of plastic pollution in surface waters around Japan in the western North Pacific over the course of 70 years (1949–2020). (a) Frequency of occurrence and (b) mean number of pieces per km^2^. The figure portrays a 1° × 1° grid with a 10-year time interval. Five-pointed star in the 1950s panel indicates the location where the earliest plastic debris was collected in 1952.

**Figure 2:**
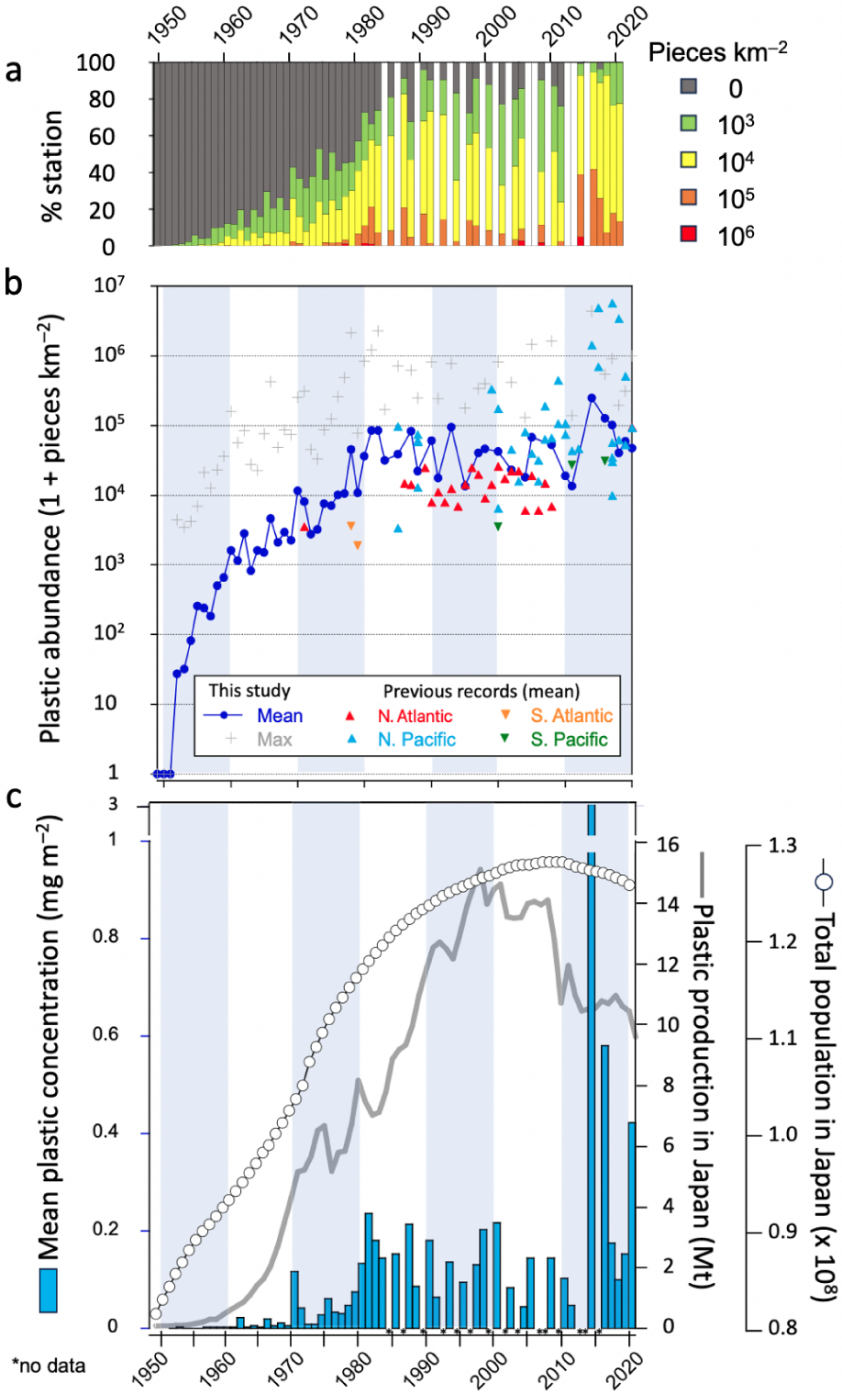
Temporal changes in plastic pollution in surface waters around Japan in the western North Pacific over the course of 70 years (1949–2020). (a) Composition of the number of stations, in terms of plastic abundance (pieces/km^2^), and (b) mean and maximum abundance of plastic debris in surface waters (pieces/km^2^). Records from previous studies (data sources are shown in Table S2). (c) Mean concentration of plastic debris in surface waters (mg/km^2^). Mean weights were estimated from plastic debris size; length-weight allometric equations were established depending on the morphotypes (see Figure S3). Historical changes in the total plastic production and total population in Japan are shown using gray lines and open circles, respectively.

No plastic debris was detected in the samples collected between 1949 and 1951 in Japan. However, the earliest instance of plastic debris in the OSL in Japan was observed in 1952, off the coast of Aomori and north of Honshu, which is the largest island of Japan. The occurrence and abundance of plastic debris in the OSL showed an increasing trend, beginning from the Pacific coast of northeastern Japan in the 1950s and expanding to the Pacific coast of western Japan in the 1960s, while the occurrence rate remained at a low level (Figures 1a and 2a). Thereafter, the mean abundance of plastic debris at the OSL around Japan increased to over 800 times in the late 1970s on average, from its first occurrence in 1952, reaching a maximum of 10^5^ pieces/km^2^ (Figures 1b and 2b). The rate of increase began settling in the 1980s, remaining between 10^4^ and 10^5^ pieces/km^2^ until the 2000s. However, since the mid-2010s, the number density has shown a tendency to increase. Several years portrayed an average of >10^5^ pieces/km^2^. The weight concentration of the plastic debris exhibited a pattern similar to that of plastic abundance (Figure 2b). Between the 1950s and 1970s, the mean concentration steadily increased from an average of 0.001 to 0.045 mg/m^2^. In the 1980s, it sharply increased to 0.163 mg/m^2^ and then remained at the same level until the mid-2010s, although a few years portrayed values exceeding 0.5 mg/m^2^.

**Figure 3:**
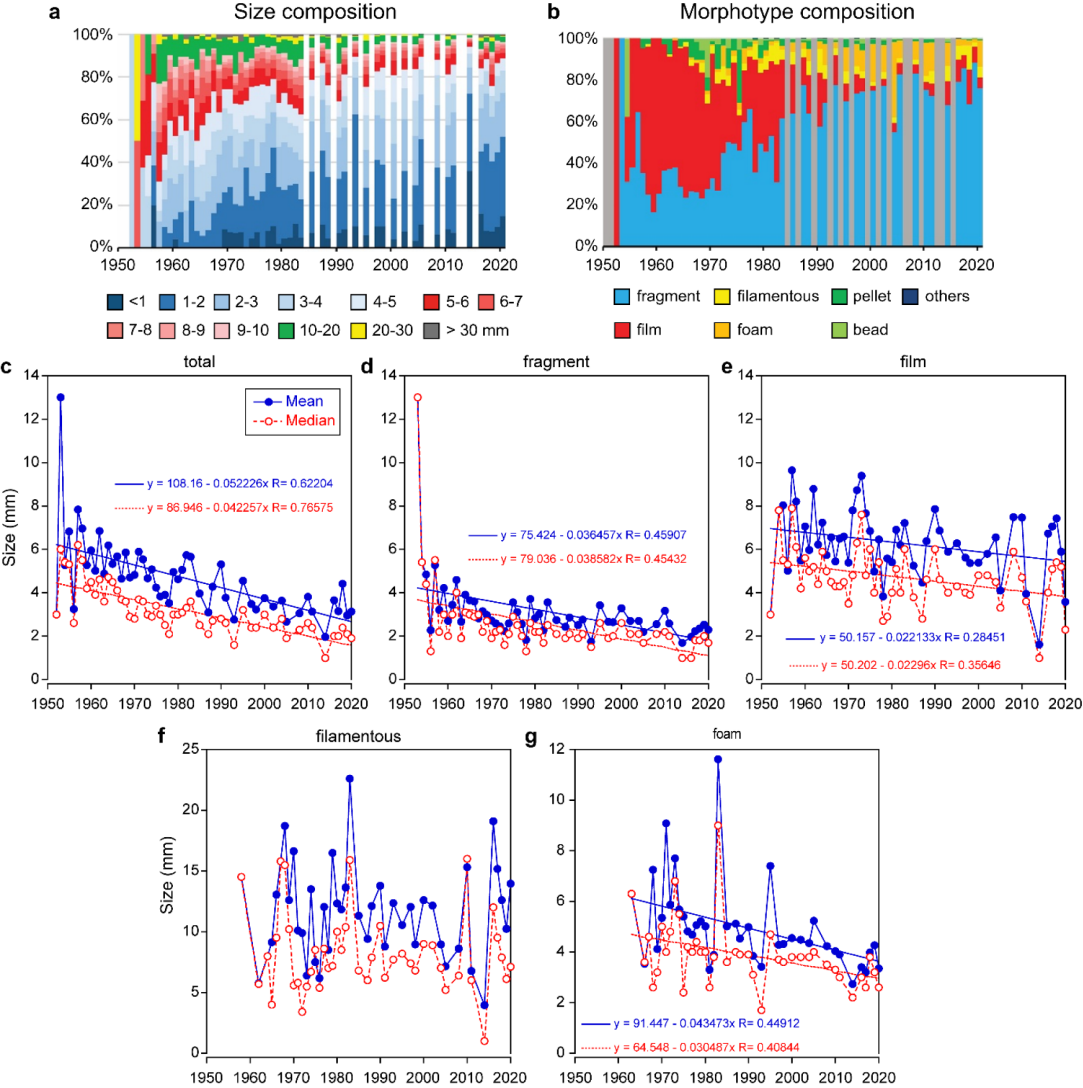
Temporal change in the qualitative properties of plastic debris in the surface waters around Japan in the western North Pacific over the course of 70 years (1949–2020). (a) Size and (b) morphotype compositions of the debris by particle count (weight compositions were also shown in Figure S5); temporal changes in the mean and median size of c) total, d) fragment, e) film, f) filamentous, and g) foam plastic debris.

We noted temporal changes in the size composition of the plastic debris; generally, plastics <10 mm made up >80% of the total plastic particles recorded during the study period. However, the proportion of microplastics <5 mm has increased over time, consistently accounting for 80% of the total number of particles collected after the 1990s (Figure 3a). Notably, our analysis revealed a change in the shape of the plastic debris over time. From the 1950s to the 1970s, film plastics accounted for half of the total number of pieces. However, from the 1980s onward, the plastic debris at the OSL mainly consisted of fragments, accounting for 60–80% of total debris (Figure 3b). Additionally, the annual mean and median sizes of all plastic particles decreased significantly over time. In the late 1960s and 1970s, the proportion of primary plastics (beads and pellets) was high, accounting for up to 20–30% of the total collected debris in number and 40–50% in weight (Figures 3b and S4). Since the 1980s, the contribution of primary plastics has remained below 5% of the total in number and 10% in weight (Figure S5). Notably, our study confirms that the contribution of foam plastic has steadily increased since the 1980s, accounting for up to 40% of the plastic debris in the OSL. At the onset of the discharge of plastic into oceans, the mean size of all plastic debris in the OSL was approximately 6 mm; this has currently reduced to 3 mm, portraying a decrease in the size by half over the 70 years (Figure 3c). The mean and median sizes of the three main types of plastics decreased significantly over time; however, this was not the case for filamentous plastics (Figure 3d–g).

## 4 Discussion

Our results suggest that the amount of plastic debris in the OSL in the WNP has steadily increased since the beginning of plastic discharge in 1952, and this increase has not been monotonic. The temporal changes in plastic debris in the OSL in the WNP can be classified into three distinct periods, i.e., 1) a period of increase from the 1950s to the late 1970s; 2) a stagnation period, with a high plastic-debris density, from the 1980s to the early 2010s; and 3) a period of reincrease after mid-2010s.

Our analysis indicates that plastic pollution in the WNP began in the early 1950s. The plastic collected in 1952 in the North Pacific predated the earliest record of marine plastic debris collected in the field in 1957 in the North Atlantic Ocean^13^ and precisely coincided with the beginning of the Anthropocene, recently defined by Kuwae et al.^30^ The data set collated in this study indicated that during the 1950s, the area contaminated by plastic waste initially expanded off the coast of Tohoku (in northeastern Japan), probably due to the transport of the debris by the Kuroshio and Tsugaru Warm Currents and the convergence effect of anticyclonic eddies.^31^ In the 1960s, the areas contaminated by plastic debris extended to the western coastal areas of Japan; there was still a substantial area with no plastic occurrence, particularly in the offshore area (Figures 1b and 2a). This suggests that during the early phase of marine plastic pollution in the region, the main source of contamination was within Japan, as indicated by the exponential increase in domestic plastic production in the country during the 1950s–1960s (Figure 2c). Subsequently, the areas contaminated by plastic debris spread offshore; by the late 1970s, plastic debris was detected in >50% of the samples collected in the region, and the mean abundance reached an order of 10^4^ pieces/km^2^.

Since the late 1970s, a clear increasing trend in the abundance of plastic debris was not noted until the mid-2010s, with values fluctuating between 10^4^ and 10^5^ pieces/km^2^, despite the continuous increase in domestic plastic production by 1997 as well as in the total population in Japan by 2008 (Figure 2b,c). Notably, the abundance of plastic debris during this period is largely consistent with previous reports of the North Pacific (Figure 2B). Furthermore, the records from the North Atlantic for the same period reported a stable plastic abundance of approximately 10^4^ pieces/km^2^,^32^ suggesting that the stagnating trend during this period may be a globally observed phenomenon.^17,18^ It has been suggested that the implementation of various global policy measures in several countries since the 1970s partially explains this stagnation trend,^18^ although it has not been verified quantitatively whether the measures have been sufficient to reduce the increase in marine plastics. In Japan, the Waste Disposal and Public Cleansing Law was first enacted in 1970. It was revised ten times before 2010 to strengthen the waste management system, which included waste reduction and recycling.^33^ The Container and Packaging Recycling Law, which included the establishment of a plastic recycling system, was enacted in 1995.^33^ This legislation may have reduced the discharge of plastic from Japan into the surrounding oceans to some extent. However, in the case of the western North Pacific, it is unlikely that the measures in Japan effectively suppressed the ocean plastic abundance until the mid-2010s when economic growth in East Asia has already started.

Our results indicated that the onset of the stagnation period corresponded to the time when the presence of plastics in the collected samples exceeded 80%, and its distribution expanded far offshore (Figures 1a and 2a). During this period, there was a shift in the dominant shape of plastics from film-shaped plastics, which could be easily removed in coastal areas,^34,35^ to fragment-shaped plastics, which portrayed higher buoyancy. Additionally, a progressive decrease in size mitigated the impact of the nearshore trapping process on larger-sized plastics that were selectively transported onshore by a combined process involving Stokes drift and terminal velocity.^36^ The effects described may have contributed to the offshore dispersal of plastic debris from the late 1970s onward, resulting in density dilution and, consequently, a reduction in the apparent increase in the study area.

**Figure 4:**
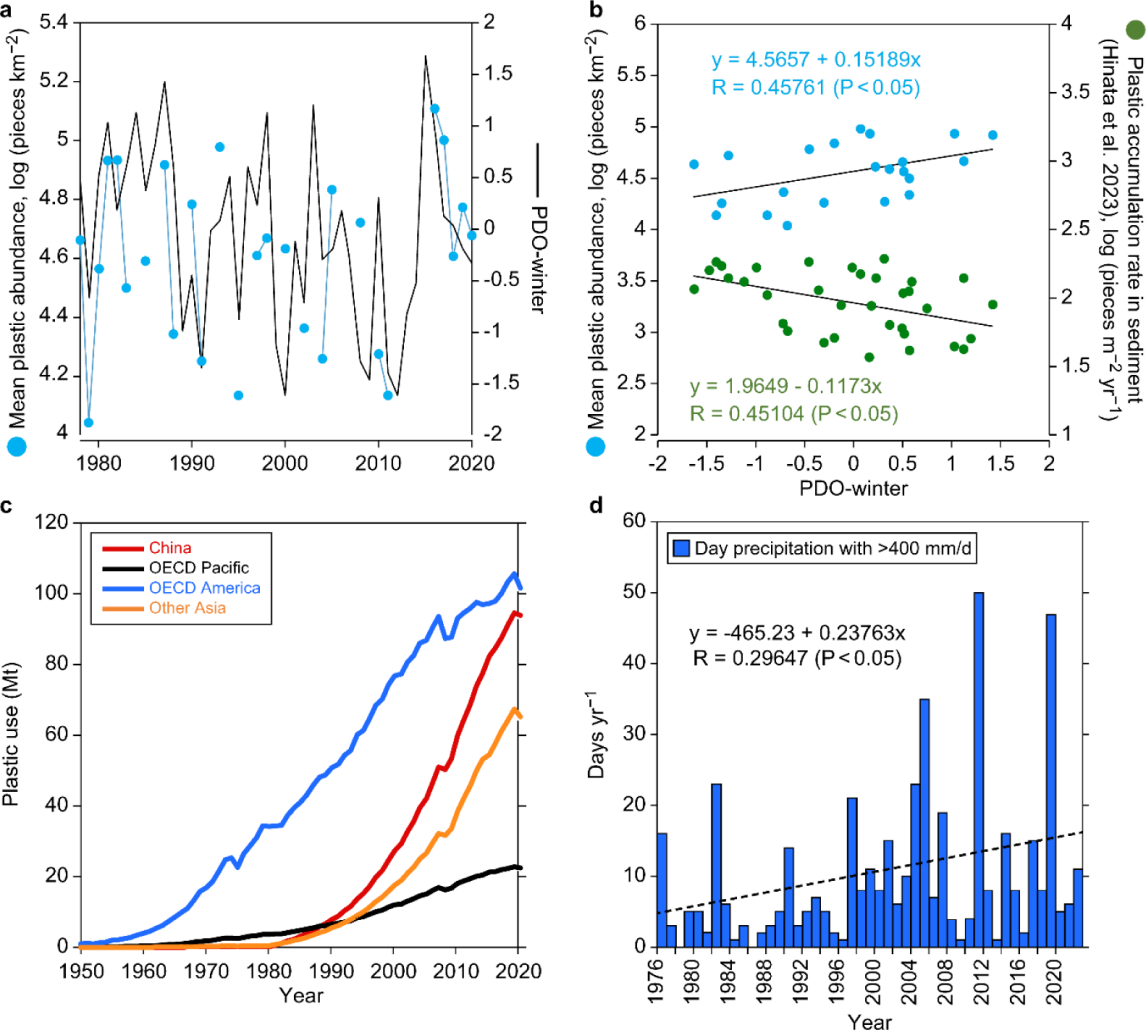
Temporal changes in plastic abundance and the potential driving factors. (a) Mean abundance of plastic debris around Japan and winter PDO index during 1978–2022; (b) relationships between winter Pacific decadal oscillation (PDO) index and the mean abundance of plastic debris during the stagnant period (1978–2011) around Japan (blue dots) and plastic-accumulation rate derived from the sediment core of Beppu Bay (green dots).^33^ (c) Historical changes in annual plastic use in the Organization for Economic Cooperation and Development (OECD) Pacific region (Japan, Korea, Australia, and New Zealand), OECD American area (USA, Canada, Mexico, Chile, Colombia, and Costa Rica), China, and non-OECD Asian countries. (d) Recent changes in the number of days of heavy rainfall (400 mm/day) and its trends during 1976–2022 in Japan.

The expansion of the distribution area of plastic debris offshore possibly resulted in an increase in the area requiring plastic removal. Further, a decrease in plastic debris size may result in an increase in the fraction of sediment requiring plastic removal and also contribute to the stabilization of the abundance of the plastic debris in the OSL, given that small-sized microplastics, with low buoyancy, can be rapidly eliminated from the OSL as aggregates by phytoplankton and via biofilm formation.^37,38^ To investigate the impact of biological production on sediment removal, we compared the abundance of plastic debris in the OSL with the winter Pacific decadal oscillation (PDO) index, which reflects the anomalies in hydrographic conditions in the North Pacific.^39^ Previous studies in the WNP have indicated that nutrient contents decline as PDO signals increase,^40,41^ and a strong negative correlation between the winter PDO index and the annual net primary production was reported.^42^ The comparison revealed a significant positive correlation (*p* < 0.05) between the two during the stagnation period (1978–2011; Figure 4a,b). Furthermore, the winter PDO index for the same period exhibited a significant positive relationship with the total amount of plastic debris removed during 1 year from the water column in Beppu Bay in western Japan, located near the study site.^29^ Hinata et al.^29^ proved that the sedimented plastic debris in Beppu Bay was significantly related to the chlorophyll*-a* concentration in the sediments, indicating that they were largely removed by phytoplankton aggregation. These observations suggested that during stagnation in the OSL in the WNP, annual variations in primary production considerably affected plastic debris abundance, possibly via a combination of several processes, including sedimentation, phytoplankton aggregation, and grazing by gelatinous zooplankton.^43,44^ Pelagic tunicates, such as salps and doliolids, are known to reproduce asexually and form blooms (in response to sporadic increases in primary production) and occur frequently in the study area.^45^ They ingest suspended particles nonselectively through filter feeding (including plastics smaller than 1 mm)^41^ and produce large fecal pellets with a high sinking rate.^43^ Therefore, they play a significant role in the efficient removal of microplastics with high primary production.

In the mid-2010s, the density of plastic debris in the OSL reincreased abruptly, which could be attributed to the increase in the number of stations with extremely high plastic debris densities (≥10^5^ pieces/km^2^) and the disappearance of stations with no plastic debris (Figure 2a), indicating that plastic pollution spread over the entire ocean. Although the density of floating plastic debris during the combined “stagnant” and “re-increase” period exhibited a significant correlation with the winter PDO index, the correlation coefficient exhibited a decrease (*R* = 0.3746, *n* = 28, *p* < 0.05), suggesting that a different mechanism was involved in the increase in plastic abundance (compared to that observed in the previous period). There have been reports of an abrupt increase in floating plastic debris in the OSL since 2000 in various areas, including the North Atlantic,^13^ western North Atlantic subtropical gyre,^20^ and the Great Pacific Garbage Patch,^19^ which could be attributed to the global increase in plastic production.^18^ In recent years, plastic production has increased dramatically in the Pacific Rim countries, especially in China and non-Organization for Economic Cooperation and Development (OECD) Asian countries, including Indonesia, the Philippines, and Vietnam, where plastic production has far surpassed that of Japan since the 1990s (Figure 4c); notably, these countries have severe issues related to the mismanagement of plastic waste.^46^ Thus, increased plastic discharge from these countries into the ocean may have contributed to the increase in plastic debris in the study area, which is located downstream of the coastal areas of these countries. Furthermore, the increase in plastic debris may have resulted from the increase in natural disasters in recent years. On March 11, 2011, the Tohoku tsunami released more than 13 times the total amount of plastic contained in the Atlantic Ocean into the Pacific Ocean in a single event, and the plastic spread to the WNP within a year.^47^ Furthermore, a recent increase in extreme precipitation (Figure 4d), partly due to climate change,^48,49^ which causes a remarkably high abundance of plastic debris on a local scale due to the enhancement of the loading of plastic debris through river inflow,^50^ may have contributed to the reincrease trend to some extent. A decrease in the primary productivity associated with the recent increase in water temperature has been reported in the WNP;^42,51^ a decrease in the sediment-removal potential by phytoplankton aggregation may also have contributed to the increase in plastic debris.

This study is the first to show that all morphotypes of floating plastic debris, except filamentous debris, have continuously decreased in size from the onset of plastic discharge to the present day. Plastics floating on the OSL gradually degrade due to various factors, including heat, humidity, UV radiation, reactions with ozone and chemicals, mechanical forces, and microorganisms;^52^ however, as most of them are generally removed from the surface layer in the WNP within 3–5 years,^53,54^ it is unlikely that the size of plastic debris could become smaller over a long period of time. Therefore, the successive decrease in the size of the plastic debris observed in this study indicates two possibilities: first, the fraction of smaller particles discharged into the ocean has increased over time; second, the environment in the ocean is evolving to facilitate the miniaturization of the plastic debris in the OSL. The former may entail historical changes with respect to the supply process. A recent study on the global ocean surface mass balance budget for positively buoyant macroplastic suggests that there is a significant time interval, ranging from several years to decades, between terrestrial emissions and the representative accumulation of ocean plastics in offshore waters.^55^ The majority of secondary microplastics found in the world’s oceans are from items produced in the 1990s or earlier; these plastics continue to degrade to date.^55^ Thus, the current source of floating plastic debris is a reservoir with degraded plastic, e.g., beaches, estimated to accumulate 27.3% of the total ocean plastic.^5^ Because the beach is an efficient generator of microplastics,^56^ its increased contribution to the oceanic emissions of plastic debris may explain the successive decrease in the size of the plastic debris in the OSL. In the latter case, long-term changes in marine biota, known to interact with plastic debris, could be involved in the size-selective utilization/degradation/removal of large plastic debris.^57−59^ Furthermore, the ongoing period of global warming,^60^ characterized by accelerated photodegradation of plastic debris owing to enhanced surface stratification,^61^ could contribute to miniaturization of plastic debris in both scenarios. Notably, an increase in the frequency and intensity of tropical cyclones owing to climate change^48,49^ enhances the fragmentation of plastic debris at beaches and in the OSL. Furthermore, the progressive decrease in the size of microplastics over time may increase the likelihood of their contamination of the food chain, potentially posing a greater risk to ecosystems^10,12^ while enhancing the plastic-removal process.

This study is also the first to provide empirical evidence that the concentration of floating plastic debris in the OSL did, in fact, exhibit stagnation for >35 years, which has been a subject of debate in the literature,^17,18^ and thereafter increased abruptly within the past decade. Overall, the long-term changes in the distribution of plastic debris in the OSL around Japan align closely with previously reported patterns observed within shorter periods across different locations; this finding further implies a consistent trend in the global distribution of plastic debris. Although the results of this study are limited to the OSL of the WNP, when considered alongside the results of modeling studies and surveys of water column and seafloor sediment distribution, they contribute to a greater understanding of the past and future dynamics of plastic pollution throughout the North Pacific. Further, we demonstrated that marine plastic pollution is influenced by complex factors, including marine productivity, climate change, human-related factors, and marine biota response. Thus, collaborations across different disciplines are required to provide a comprehensive understanding of the dynamics of marine plastic pollution. The recent surge in floating marine plastic debris strongly suggests that plastic discharge is outpacing its removal capacity, suggesting that the effect of the pollution on the epipelagic ecosystems is now growing and expanding. A similar trend has been identified in other regions worldwide;^18^ therefore, it is likely to be closely linked to environmental issues that are progressing synchronously across the globe, i.e., increased plastic waste and global warming, which directly affect the ocean environment. Therefore, the problem of plastic pollution necessitates a coordinated approach incorporating effective measures to combat climate change through collaboration among various stakeholders.

## Supporting Information Available

Summary of the information related to the samples analyzed in this study (Table S1); records of plastic abundance in the ocean surface layer shown in Figure 2B (Table S2); location of sampling stations used in this study (Figure S1); procedure for the analysis of plastic debris (Figure S2); data used for intercalibration of two types of plankton nets used during the early phase of monitoring (Figure S3); length-weight allometric equations used to estimate the plastic concentration (mg/m²) for Figure 2C (Figure S4); temporal change in the weight compositions of the plastic debris collected in the surface waters around Japan in the western North Pacific over the course of 70 years (1949–2020) (Figure S5) (PDF)

## References

[ref1] Eriksen M.; Lebreton L. C. M.; Carson H. S.; Thiel M.; Moore C. J.; Borerro J. C.; Galgani F.; Ryan P. G.; Reisser J. Plastic pollution in the world’s oceans: More than 5 trillion plastic pieces weighing over 250,000 tons afloat at sea. PLoS One 2014, 9, e111913 10.1371/journal.pone.0111913. 25494041 PMC4262196

[ref2] Wayman C.; Niemann H. The fate of plastic in the ocean environment–a mini review. Environ. Sci. Process Impacts 2021, 23, 198–212. 10.1039/D0EM00446D. 33475108

[ref3] Kaandorp M. L. A.; Lobelle D.; Kehl C.; Dijkstra H. A.; van Sebille E. Global mass of buoyant marine plastics dominated by large long-lived debris. Nat. Geosci. 2023, 16, 689–694. 10.1038/s41561-023-01216-0.

[ref4] Weiss L.; Ludwig W.; Heussner S.; Canals M.; Ghiglione J. F.; Estournel C.; Constant M.; Kerhervé P. The missing ocean plastic sink: Gone with the rivers. Science 2021, 373, 107–111. 10.1126/science.abe0290. 34210886

[ref5] Isobe A.; Iwasaki S. The fate of missing ocean plastics: Are they just a marine environmental problem?. Sci. Total Environ. 2022, 825, 153935 10.1016/j.scitotenv.2022.153935. 35192833

[ref6] Choy C. A.; Robison B. H.; Gagne T. O.; Erwin B.; Firl E.; Halden R. U.; Hamilton J. A.; Katija K.; Lisin S. E.; Rolsky C.; Van Houtan K. S. The vertical distribution and biological transport of marine microplastics across the epipelagic and mesopelagic water column. Sci. Rep. 2019, 9, 7843 10.1038/s41598-019-44117-2. 31171833 PMC6554305

[ref7] Egger M.; Sulu-Gambari F.; Lebreton L. First evidence of plastic fallout from the North Pacific Garbage Patch. Sci. Rep. 2020, 10, 7495 10.1038/s41598-020-64465-8. 32376835 PMC7203237

[ref8] Gerritse J.; Leslie H. A.; de Tender C. A.; Devriese L. I.; Vethaak A. D. Fragmentation of plastic objects in a laboratory seawater microcosm. Sci. Rep. 2020, 10, 10945 10.1038/s41598-020-67927-1. 32616793 PMC7331685

[ref9] Li W. C.; Tse H. F.; Fok L. Plastic waste in the marine environment: A review of sources, occurrence and effects. Sci. Total Environ. 2016, 566–567, 333–349. 10.1016/j.scitotenv.2016.05.084. 27232963

[ref10] Hale R. C.; Seeley M. E.; La Guardia M. J.; Mai L.; Zeng E. Y. A global perspective on microplastics. JGR Oceans 2020, 125, e2018JC014719 10.1029/2018JC014719.

[ref11] Harb C.; Pokhrel N.; Foroutan H. Quantification of the emission of atmospheric microplastics and nanoplastics via sea spray. Environ. Sci. Technol. Lett. 2023, 10, 513–519. 10.1021/acs.estlett.3c00164.

[ref12] Cole M.; Lindeque P.; Halsband C.; Galloway T. S. Microplastics as contaminants in the marine environment: A review. Mar. Pollut. Bull. 2011, 62, 2588–2597. 10.1016/j.marpolbul.2011.09.025. 22001295

[ref13] Ostle C.; Thompson R. C.; Broughton D.; Gregory L.; Wootton M.; Johns D. G. The rise in ocean plastics evidenced from a 60-year time series. Nat. Commun. 2019, 10, 1622 10.1038/s41467-019-09506-1. 30992426 PMC6467903

[ref14] Carpenter E. J.; Smith Jr K. L. Plastics on the sargasso sea surface. Science 1972, 175, 1240–1241. 10.1126/science.175.4027.1240. 5061243

[ref15] Law K. L.; Morét-Ferguson S.; Maximenko N. A.; Proskurowski G.; Peacock E. E.; Hafner J.; Reddy C. M. Plastic accumulation in the North Atlantic subtropical gyre. Science 2010, 329 (5996), 1185–1188. 10.1126/science.1192321. 20724586

[ref16] Day R.; Shaw D. G.; Ignell S. The quantitative distribution and characteristics of neuston plastic in the North Pacific Ocean, 1984–1988; Marine Debris NOAA, Hawaii Publisher, 1990; pp. 247–266.

[ref17] Galgani F.; Brien A. S.-O; Weis J.; Ioakeimidis C.; Schuyler Q.; Makarenko I.; Griffiths H.; Bondareff J.; Vethaak D.; Deidun A.; et al. Are litter, plastic and microplastic quantities increasing in the ocean?. Micropl. Nanopl. 2021, 1 (1), 1–4. 10.1186/s43591-020-00002-8.

[ref18] Eriksen M.; Cowger W.; Erdle L. M.; Coffin S.; Villarrubia-Gómez P.; Moore C. J.; Carpenter E. J.; Day R. H.; Thiel M.; Wilcox C. A growing plastic smog, now estimated to be over 170 trillion plastic particles afloat in the world’s oceans—Urgent solutions required. PLoS One 2023, 18, e0281596 10.1371/journal.pone.0281596. 36888681 PMC9994742

[ref19] Lebreton L.; Slat B.; Ferrari F.; Sainte-Rose B.; Aitken J.; Marthouse R.; Hajbane S.; Cunsolo S.; Schwarz A.; Levivier A.; Noble K.; Debeljak P.; Maral H.; Schoeneich-Argent R.; Brambini R.; Reisser J. Evidence that the Great Pacific Garbage Patch is rapidly accumulating plastic. Sci. Rep. 2018, 8, 4666 10.1038/s41598-018-22939-w. 29568057 PMC5864935

[ref20] Wilcox C.; Hardesty B. D.; Law K. L. Abundance of floating plastic particles is increasing in the western North Atlantic Ocean. Environ. Sci. Technol. 2020, 54, 790–796. 10.1021/acs.est.9b04812. 31738052

[ref21] Ogi H.; Fukumoto Y. A sorting method for small plastic debris floating on the sea surface and stranded on sandy beaches, Bull. Fac. Fish. Hokkaido Univ., 2000, 51, 71–93

[ref22] Suaria G.; Achtypi A.; Perold V.; Lee J. R.; Pierucci A.; Bornman T. G.; Aliani S.; Ryan P. G. Microfibers in oceanic surface waters: A global characterization. Sci. Adv. 2020, 6 (23), eaay8493 10.1126/sciadv.aay8493. 32548254 PMC7274779

[ref23] Nakai Z. Apparatus for collecting macroplankton in the spawning surveys of iwashi (sardine, anchovy, and round herring) and others, Bull. Tokai Reg. Fish. Res. Lab., 1962, 9, 221–237.

[ref24] Oozeki Y. Comparison of catch efficiencies between the Manta net and surface ring net for sampling larvae and juveniles of Pacific saury, Cololabis saira, Bull. Jpn. Soc. Fish. Oceanogr., 2000, 64, 18–24.

[ref25] Oozeki Y.; Kimura R.; Kubota H.; Ishida M. Modified neuston net for collecting larvae and juveniles of Pacific saury, Cololabis Saria. Bull. Jpn. Soc. Fish. Oceanogr., 2001, 65, 1, 5.

[ref26] Brown D. M.; Cheng L. New net for sampling the ocean surface. Mar. Ecol.: Prog. Ser. 1981, 5, 225–227. 10.3354/meps005225.

[ref27] Takasuka A.; Nyuji M.; Kuroda H.; Oozeki Y. Variability of swept area by sea-surface tows of a neuston net: Balance of resistance, clogging, and over-inflow effects. Fish. Res. 2019, 210, 175–180. 10.1016/j.fishres.2018.10.021.

[ref28] Tokai T.; Uchida K.; Kuroda M.; Isobe A. Mesh selectivity of neuston nets for microplastics. Mar. Pollut. Bull. 2021, 165, 112111 10.1016/j.marpolbul.2021.112111. 33588104

[ref29] Hinata H.; Kuwae M.; Tsugeki N.; Masumoto I.; Tani Y.; Hatada Y.; Kawamata H.; Mase A.; Kasamo K.; Sukenaga K.; Suzuki Y. A 75-year history of microplastic fragment accumulation rates in a semi-enclosed hypoxic basin. Sci. Total Environ. 2023, 854, 158751 10.1016/j.scitotenv.2022.158751. 36113797

[ref30] Kuwae M.; Yokoyama Y.; Tims S.; Froehlich M.; Fifield L. K.; Aze T.; Tsugeki N.; Doi H.; Saito Y. Toward defining the Anthropocene onset using a rapid increase in anthropogenic fingerprints in global geological archives. Proc. Natl. Acad. Sci. U. S. A. 2024, 121 (41), e2313098121 10.1073/pnas.2313098121. 39312679 PMC11474069

[ref31] Miyazono K.; Yamashita R.; Miyamoto H.; Ishak N. H. A.; Tadokoro K.; Shimizu Y.; Takahashi K. Large-scale distribution and composition of floating plastic debris in the transition region of the North Pacific. Mar. Pollut. Bull. 2021, 170, 112631 10.1016/j.marpolbul.2021.112631. 34175698

[ref32] Law K. L.; Morét-Ferguson S. E.; Goodwin D. S.; Zettler E. R.; DeForce E.; Kukulka T.; Proskurowski G. Distribution of surface plastic debris in the eastern Pacific Ocean over an 11-year dataset. Environ. Sci. Technol. 2014, 48, 4732–4738. 10.1021/es4053076. 24708264

[ref33] Hara K.; Yabar H. Historical evolution and development of waste management and recycling systems—Analysis of Japan’s experiences. J. Environ. Stud. Sci. 2012, 2, 296–307. 10.1007/s13412-012-0094-8.

[ref34] Isobe A.; Kubo K.; Tamura Y.; Kako S.; Nakashima E.; Fujii N. Selective transport of microplastics and mesoplastics by drifting in coastal waters. Mar. Pollut. Bull. 2014, 89, 324–330. 10.1016/j.marpolbul.2014.09.041. 25287228

[ref35] Ryan P. G. Does size and buoyancy affect the long-distance transport of floating debris?. Environ. Res. Lett. 2015, 10, 084019 10.1088/1748-9326/10/8/084019.

[ref36] Isobe A.; Uchida K.; Tokai T.; Iwasaki S. East Asian seas: A hot spot of pelagic microplastics. Mar. Pollut. Bull. 2015, 101, 618–623. 10.1016/j.marpolbul.2015.10.042. 26522164

[ref37] Kaiser D.; Kowalski N.; Waniek J. J. Effects of biofouling on the sinking behavior of microplastics. Environ. Res. Lett. 2017, 12, 124033 10.1088/1748-9326/aa8e8b.

[ref38] Michels J.; Stippkugel A.; Lenz M.; Wirtz K.; Engel A. Rapid aggregation of biofilm-covered microplastics with marine biogenic particles. Proc. Biol. Sci. 2018, 285, 20181203 10.1098/rspb.2018.1203. 30158309 PMC6125906

[ref39] Minobe S. Spatio-temporal structure of the pentadecadal variability over the North Pacific. Prog. Oceanogr. 2000, 47, 381–408. 10.1016/S0079-6611(00)00042-2.

[ref40] Limsakul A.; Saino T.; Midorikawa T.; Goes J. I. Temporal variations in low trophic level biological environments in the northwestern North Pacific Subtropical gyre from 1950 to 1997. Prog. Oceanogr. 2001, 49, 129–149. 10.1016/S0079-6611(01)00019-2.

[ref41] Aita M. N.; Yamanaka Y.; Kishi M. J. Interdecadal variation of the lower trophic ecosystem in the northern Pacific between 1948 and 2002, in a 3-D implementation of the NEMURO model. Ecol Modell. 2007, 202, 81–94. 10.1016/j.ecolmodel.2006.07.045.

[ref42] Watanabe Y. W.; Ishida H.; Nakano T.; Nagai N. Spatiotemporal decreases of nutrients and chlorophyll-a in the surface mixed layer of the western North Pacific from 1971 to 2000. J. Oceanogr. 2005, 61, 1011–1016. 10.1007/s10872-006-0017-y.

[ref43] Wieczorek A. M.; Croot P. L.; Lombard F.; Sheahan J. N.; Doyle T. K. Microplastic ingestion by gelatinous zooplankton may lower efficiency of the biological pump. Environ. Sci. Technol. 2019, 53, 5387–5395. 10.1021/acs.est.8b07174. 30932485

[ref44] Sutherland K. R.; Madin L. P.; Stocker R. Filtration of submicrometer particles by pelagic tunicates. Proc. Natl. Acad. Sci. U. S. A. 2010, 107, 15129–15134. 10.1073/pnas.1003599107. 20696887 PMC2930554

[ref45] Ishak N. H. A.; Tadokoro K.; Okazaki Y.; Kakehi S.; Suyama S.; Takahashi K. Distribution, biomass and species composition of salps and doliolids in the Oyashio-Kuroshio Transitional Region: Potential impact of massive bloom on the pelagic food web. J. Oceanogr. 2020, 76, 351–363. 10.1007/s10872-020-00549-3.

[ref46] Jambeck J. R.; Geyer R.; Wilcox C.; Siegler T. R.; Perryman M.; Andrady A.; Narayan R.; Law K. L. Marine pollution. Plastic waste inputs from land into the ocean. Science 2015, 347, 768–771. 10.1126/science.1260352. 25678662

[ref47] Lebreton L. C. M.; Borrero J. C. Modeling the transport and accumulation floating debris generated by the 11 March 2011 Tohoku tsunami. Mar. Pollut. Bull. 2013, 66, 53–58. 10.1016/j.marpolbul.2012.11.013. 23219397

[ref48] Knutson T. R.; McBride J. L.; Chan J.; Emanuel K.; Holland G.; Landsea C.; Held I. M.; Kossin J. P.; Srivastava A. K.; Sugi M. Tropical cyclones and climate change. Nat. Geosci. 2010, 3, 157–163. 10.1038/ngeo779.

[ref49] Kossin J. P.; Knapp K. R.; Olander T. L.; Velden C. S. Global increase in major tropical cyclone exceedance probability over the past four decades. Proc. Natl. Acad. Sci. U. S. A. 2020, 117, 11975–11980. 10.1073/pnas.1920849117. 32424081 PMC7275711

[ref50] Nakajima R.; Miyama T.; Kitahashi T.; Isobe N.; Nagano Y.; Ikuta T.; Oguri K.; Tsuchiya M.; Yoshida T.; Aoki K.; Maeda Y.; Kawamura K.; Suzukawa M.; Yamauchi T.; Ritchie H.; Fujikura K.; Yabuki A. Plastic after an extreme storm: The typhoon-induced response of micro-and mesoplastics in coastal waters. Front Mar Sci. 2022, 8, 806952 10.3389/fmars.2021.806952.

[ref51] Ishida H.; Watanabe Y. W.; Ishizaka J.; Nakano T.; Nagai N.; Watanabe Y.; Shimamoto A.; Maeda N.; Magi M. Possibility of recent changes in vertical distribution and size composition of chlorophyll a in the western North Pacific Region. J. Oceanogr. 2009, 65, 179–186. 10.1007/s10872-009-0017-9.

[ref52] Maddison C.; Sathish C. I.; Lakshmi D.; Wayne O.; Palanisami T. An advanced analytical approach to assess the long-term degradation of microplastics in the marine environment. NPJ. Mater. Degrad. 2023, 7, 59 10.1038/s41529-023-00377-y.

[ref53] Isobe A.; Iwasaki S.; Uchida K.; Tokai T. Abundance of non-conservative microplastics in the upper ocean from 1957 to 2066. Nat. Commun. 2019, 10, 417 10.1038/s41467-019-08316-9. 30679437 PMC6345988

[ref54] Okubo R.; Yamamoto A.; Kurima A.; Sakabe T.; Ide Y.; Isobe A. Estimation of the age of polyethylene microplastics collected from oceans: Application to the western North Pacific Ocean. Mar. Pollut. Bull. 2023, 192, 114951 10.1016/j.marpolbul.2023.114951. 37172339

[ref55] Lebreton L.; Egger M.; Slat B. A global mass budget for positively buoyant macroplastic debris in the ocean. Sci. Rep. 2019, 9, 12922 10.1038/s41598-019-49413-5. 31515537 PMC6742645

[ref56] Kalogerakis N.; Karkanorachaki K.; Kalogerakis G. C.; Triantafyllidi E. I.; Gotsis A. D.; Partsinevelos P.; Fava F. Microplastics generation: Onset of fragmentation of polyethylene films in marine environment mesocosms. Front Mar Sci. 2017, 4, 00084 10.3389/fmars.2017.00084.

[ref57] Dawson A. L.; Kawaguchi S.; King C. K.; Townsend K. A.; King R.; Huston W. M.; Nash S. M. B. Turning microplastics into nanoplastics through digestive fragmentation by Antarctic krill. Nat. Commun. 2018, 9, 1001 10.1038/s41467-018-03465-9. 29520086 PMC5843626

[ref58] Santos R. G.; Machovsky-Capuska G. E.; Andrades R. Plastic ingestion as an evolutionary trap: Toward a holistic understanding. Science 2021, 373, 56–60. 10.1126/science.abh0945. 34210877

[ref59] Haram L. E.; Carlton J. T.; Centurioni L.; Choong H.; Cornwell B.; Crowley M.; Egger M.; Hafner J.; Hormann V.; Lebreton L.; Maximenko N.; McCuller M.; Murray C.; Par J.; Shcherbina A.; Wright C.; Ruiz G. M. Extent and reproduction of coastal species on plastic debris in the North Pacific Subtropical gyre. Nat Ecol Evol. 2023, 7, 687–697. 10.1038/s41559-023-01997-y. 37069334 PMC10172146

[ref60] Yang H.; Lohmann G.; Stepanek C.; Wang Q.; Huang R. X.; Shi X.; Liu J.; Chen D.; Wang X.; Zhong Y.; Yang Q.; Bao Y.; Müller J. Satellite-observed strong subtropical ocean warming as an early signature of global warming. Commun. Earth Environ. 2023, 4, 178 10.1038/s43247-023-00839-w.

[ref61] Häder D. P.; Williamson C. E.; Wängberg S. Å.; Rautio M.; Rose K. C.; Gao K.; Helbling E. W.; Sinha R. P.; Worrest R. Effects of UV radiation on aquatic ecosystems and interactions with other environmental factors. Photochem. Photobiol. Sci. 2014, 14, 108–126. 10.1039/c4pp90035a. 25388554

