# Supplementary material for: Long-Term Changes in the Abundance, Size, and Morphotype of Marine Plastics in the North Pacific
Source: Environ Sci Technol. 2025 Feb 26;59(9):4608–17. doi: 10.1021/acs.est.4c09706 (PMC11912323; doi:10.1021/acs.est.4c09706)
Supplement: Supplementary file 1 — es4c09706_si_001.pdf [file es4c09706_si_001.pdf]

Supporting Information

Long-term changes in the abundance, size, and morphotype of marine plastics in North Pacific

Kentaro Miyazono[1], Kazuaki Tadokoro[2], Gajahin G. N. Thushari[1,3], Hiroomi Miyamoto[4], Akinori Takasuka[1], Mikio Watai[4], Tohya Yasuda[4], Takuya Sato[1,5], Rei Yamashita[6], Taketoshi Kodama[1], Kazutaka Takahashi[1,*]

[1]Graduate School of Agricultural and Life Sciences, The University of Tokyo, 1-1-1, Yayoi, Bunkyo, Tokyo, Japan

[2]Japan Fisheries Research and Education Agency, 3-27-5 Shinhama-cho, Shiogama, Miyagi, 985-0001, Japan

[3]Department of Animal Science, Faculty of Animal Science and Export Agriculture, Uva Wellassa University, Passara road Badulla, Sri Lanka

[4]Japan Fisheries Research and Education Agency, 2-12-4, Fukuura, Kanazawa-ku, Yokohama, Kanagawa 236-8648, Japan

[5]Bioinformatics Center, Institute for Chemical Research, Kyoto University, Gokasho, Uji, Kyoto, 611-0011, Japan

[6]Atmosphere and Ocean Research Institute, The University of Tokyo, 5-1-5, Kashiwa-no-ha, Kashiwa, Chiba, Japan

[*] Corresponding author: kazutakahashi@g.ecc.u-tokyo.ac.jp

Summary: 15 pages, 2 tables, 5 figures.

Table S1. Summary of the information related to the samples analyzed in this study

Year	Month	Sampling gear	Mesh size (mm)	Sampling duration (min)	Number of station sampled	Sampling Area (m^2)
1949	7–8	Maruchi Net	Front: 2.00 Rear: 0.34	5	22	7366
1950	5–12				169	56581
1951	4,6–9				97	32476
1952	5–9				166	55577
1953	5–9				224	75062
1954	4–5,8–12				136	45332
1955	1,6–9,11				71	27788
1956	6–11				316	114033
1957	1–3,6–12				480	161374
1958	2–3,5–11				556	186149
1959	5–12				684	236437
1960	1–11				493	165057
1961	1–8,10–12				361	120863
1962	1–4,6–8,10–12				325	108810
1963	2–12				484	172690
1964	1–12				311	104123
1965	1–11				329	110484
1966	1–12				190	63679
1967	1–12				202	67630
1968	1–12				184	61603
1969	2–7,12				303	101445
1970	5,7–9,12				93	31070
1971	1–4				83	27788
1972	1–3,8–9,12				93	31137
1973	1–3				118	39507

1974	2–4,9				98	32811
1975	2–4				76	25445
1976	1–4,11				91	30467
1977	1–4				133	44529
1978	1–4,9				104	34819
1979	1–4				82	27454
1980	1–4,7–8,12				185	62340
1981	5,7				73	24440
1982	4,6–9				104	34819
1983	1,5–7,9				55	29061
1985	2–3,5–6,8–11				307	102985
1987	6–7	Maruchi Net /Shin-Chigyo Net			64	21226
1988	5–6		0.45	10	161	53836
1990	4–6	Shin-Chigyo Net			153	103721
1991	2–3				91	60666
1993	5–6				32	21427
1995	2				42	27788
1997	1–3,6,10–11				65	43189
1998	1–3,5–6				91	60532
2000	2–3,7	Shin-Chigyo Net /New neuston Net			167	131299
2002	2–3,5–6	New neuston Net			93	124885
2004	2				30	35065
2005	1–3,5–6				79	96214
2008	2–3,5–6				62	72228
2010	2				39	45587
2011	2–3				42	85078
2014	2–3				132	105934

2016	6–7		51	36114
2017	6–7		29	30095
2018	6–7		20	27245
2019	6–7		23	24287
2020	2		98	168181
Total			9362	4027832

Table S2. Records of plastic abundance in the ocean surface layer shown in Fig. 2B

Reference	Years of survey	Years plotted in Fig. 2B	Area	pieces/km^2	Max pieces/km^2
Carpenter and Smith (14)	1971	1971	NA	3537	12080
Law at al. (15)	1986	1986	NA	15000	
Law at al. (15)	1987	1987	NA	14000	
Law at al. (15)	1989	1989	NA	25000	
Law at al. (15)	1990	1990	NA	8000	
Law at al. (15)	1991	1991	NA	11000	
Law at al. (15)	1992	1992	NA	8000	
Law at al. (15)	1993	1993	NA	12500	
Law at al. (15)	1994	1994	NA	7000	
Law at al. (15)	1995	1995	NA	14000	
Law at al. (15)	1996	1996	NA	25000	
Law at al. (15)	1997	1997	NA	20000	580000
Law at al. (15)	1998	1998	NA	9000	
Law at al. (15)	1999	1999	NA	14000	
Law at al. (15)	2000	2000	NA	26000	
Law at al. (15)	2001	2001	NA	17500	
Law at al. (15)	2002	2002	NA	22000	
Law at al. (15)	2003	2003	NA	22000	
Law at al. (15)	2004	2004	NA	6000	
Law at al. (15)	2005	2005	NA	19000	
Law at al. (15)	2006	2006	NA	6000	
Law at al. (15)	2007	2007	NA	15000	
Law at al. (15)	2008	2008	NA	7000	
Egger et al. (61)	2020	2020	NA	95017	
Day and Shaw (62)	1985	1985	NP	96100	
Day and Shaw (62)	1985	1985	NP	3370	

Day and Shaw (62)	1985–1988	1988	NP	12800	217300
Day and Shaw (62)	1985–1988	1988	NP	57900	316800
Day and Shaw (62)	1985–1988	1988	NP	61000	
Day and Shaw (62)	1985–1988	1988	NP	74700	221000
Moore et al. (63)	1999	1999	NP	334271	969777
Uchida et al. (64)	2000–2001	2000	NP	6450	66300
Yamashita and Tanimura (65)	2000–2001	2000	NP	174000	3520000
Law et al. (66)	2002	2002	NP	45000	
Law et al. (66)	2003	2003	NP	16000	
Law et al. (66)	2004	2004	NP	82000	
Law et al. (66)	2005	2005	NP	40000	
Law et al. (66)	2006	2006	NP	16000	
Doyle et al. (67)	2006	2006	NP	31493	246600
Law et al. (66)	2007	2007	NP	63000	
Doyle et al. (67)	2007	2007	NP	189675	3140900
Law et al. (66)	2008	2008	NP	67000	
Law et al. (66)	2009	2009	NP	107000	
Goldstein et al. (68)	2009	2009	NP	448000	6553000
Law et al. (66)	2010	2010	NP	74000	
Eriksen et al. (1)	2007–2013	2010	NP	105100	
Law et al. (66)	2011	2011	NP	43000	
Law et al. (66)	2012	2012	NP	46000	
MOE, Japan (69)	2014	2014	NP	1435750	8213000

MOE, Japan (70)	2015	2015	NP	4839926	89837000
Lebreton et al. (19)	2015	2015	NP	700000	
MOE, Japan (71)	2017	2017	NP	5657758	101950000
Liu et al. (72)	2017	2017	NP	57400	99000
Mu et al. (73)	2017	2017	NP	30000	35000
Pan et al. (74)	2017	2017	NP	10000	42000
Wang et al. (75)	2017	2017	NP	34309	95335
MOE, Japan (76)	2018	2018	NP	3394428	46610000
Pan et al. (77)	2018	2018	NP	62000	220000
Pan et al. (77)	2019	2019	NP	510000	1230000
Shiu et al. (78)	2019	2019	NP	53000	150000
Xu et al. (79)	2020	2020	NP	93333	120000
Ryan (80)	1978	1978	SA	3639	445860
Morris (81)	1979	1979	SA	1900	3600
Uchida et al. (64)	2000–2001	2000	SP	3569	20300
Eriksen et al. (82)	2011	2011	SP	26898	396342
Isobe et al. (83)	2016	2016	SP	31000	99000

References

61. M. Egger, B. Schilt, H. Wolter, T. Mani, R. de Vries, E. Zettler, H. Niemann, Pelagic distribution of plastic debris (> 500 m) and marine organisms in the northern upper layer Atlantic Ocean. *Sci. Rep.* **12**, 13465 (2022). https://doi.org/10.1038/s41598-022-17742-7

62. R. H. Day, D. G. Shaw, Patterns in the abundance of pelagic plastics and tar in the North Pacific Ocean 1976–1985. *Mar. Pollut. Bull.* 18, 311–316 (1987). **https://doi.org/10.1016/S0025-326X(87)80017-6**

63. C. J. Moore, S. L. Moore, M. K. Leecaster, S. B. Weisberg, A comparison of plastics and plankton in the North Pacific central gyre. *Mar. Pollut. Bull.* 42, 1297–1300 (2001). **https://doi.org/10.1016/S0025-326X(01)00114-X**

64. K. Uchida, R. Hagita, T. Hayashi, T. Tokai, Distribution of small plastic fragments floating in the western Pacific Ocean from 2000 to 2001. *Fish. Sci,* 82, 969–974 (2016). **https://doi.org/10.1007/s12562-016-1028-2**
65. R. Yamashita, A. Tanimura, Floating plastic in the Kuroshio Current area, western North Pacific Ocean. *Mar. Pollut. Bull.* 54, 464–488 (2007). **https://doi.org/10.1016/j.marpolbul.2006.11.012**
66. K. L. Law, S. E. Morét-Ferguson, D. S. Goodwin, E. R. Zettler, E. DeForce, T. Kukulka, G. Proskurowski, Distribution of surface plastic debris in the eastern Pacific Ocean over an 11-year dataset. *Environ. Sci. Technol.* 48, 4732–4738 (2014). **https://doi.org/10.1021/es4053076**
67. M. J. Doyle, W. Watson, N. M. Bowlin, S. B. Sheavly, Plastic particles in coastal pelagic ecosystems of the Northeast Pacific Ocean. *Mar. Environ. Res.* 71, 41–52 (2011). **https://doi.org/10.1016/j.marenvres.2010.10.001**
68. M. C. Goldstein, A. J. Titmus, M. Ford, Scales of spatial heterogeneity of plastic marine debris in the Northeast Pacific Ocean. *PLOS ONE* 8, 11 (2013). **https://doi.org/10.1371/journal.pone.0080020**
69. MOE, Japan (Ministry of Environment, Japan), Research report on marine debris floating on the ocean surface and settled on the sea bed in coastal areas around Japan in 2014. (2015). **https://www.env.go.jp/water/marine_litter/26.html**
70. MOE, Japan (Ministry of Environment, Japan), Report on the 2015 commissioned survey of drifting and marine litter in offshore waters (in Japanese). (2016). **https://www.env.go.jp/content/900542842.pdf**
71. MOE, Japan (Ministry of Environment, Japan), Report on the work on the distribution of drifting and seabed litter, including drifting microplastics, and the study of indicators, etc., in FY 2008 (in Japanese). (2019). **https://www.env.go.jp/water/marine_litter/post_60.html**
72. M. Liu, Y. Ding, P. Huang, H. Zheng, W. Wang, H. Ke, F. Chen, L. Liu, M. Cai, Microplastics in the western Pacific and South China Sea: Spatial variations reveal the impact of the Kuroshio intrusion. *Environ. Pollut.* 288, 117745 (2021). **https://doi.org/10.1016/j.envpol.2021.117745**

73. J. Mu, S. Zhang, L. Qu, F. Jin, C. Fang, X. Ma, W. Zhang, J. Wang, Microplastics abundance and the characteristics of the surface waters from the Northwest Pacific, Bering Sea, and Chukchi Sea. *Mar. Pollut. Bull.* 143, 58–65 (2019). **https://doi.org/10.1016/j.marpolbul.2019.04.023**
74. Z. Pan, H. Guo, H. Chen, S. Wang, X. Sun, Q. Zou, Y. Zhang, H. Lin, S. Cai, J. Huang, Microplastics in the Northwest Pacific: Abundance, distribution, and characteristics, *Sci. Total Environ.* 650, 1913–1922 (2019). **https://doi.org/10.1016/j.scitotenv.2018.09.244**
75. S. Wang, H. Chen, X. Zhou, Y. Tian, C. Lin, W. Wang, K. Zhou, Y. Zhang, H. Lin, Microplastic abundance, distribution and composition in the mid-west Pacific Ocean. *Environ Pollut.* 264, 114125 (2020). **https://doi.org/10.1016/j.envpol.2020.114125**
76. MOE, Japan (Ministry of Environment, Japan), Report on the work on the distribution of drifting and seabed litter, including drifting microplastics, and the study of indicators, etc., FY 2019 (in Japanese). (2020). **https://www.env.go.jp/water/marine_litter/h31.html**
77. Z. Pan, Q. Liu, X. Sun, W. Li, Q. Zou, S. Cai, H. Lin, Widespread occurrence of microplastic pollution in open sea surface waters. Evidence from the mid-North Pacific Ocean. *Gondwana Research* 108, 31–40 (2021). **https://doi.org/10.1016/j.gr.2021.10.024**
78. R. F. Shiu, G. C. Gong, M. D. Fang, C.H. Chow, W.C. Chin, Marine microplastics in the surface waters of "pristine" Kuroshio. *Mar. Pollut. Bull.* 172, 112808 (2021). **https://doi.org/10.1016/j.marpolbul.2021.112808**
79. H. Xu, H. Nakano, T. Tokai, T. Miyazaki, H. Hamada, H. Arakawa, Contamination of sea surface waters offshore of the Tokai region and Tokyo Bay in Japan by small microplastics. *Mar. Pollut. Bull.* 185, 114245 (2022). **https://doi.org/10.1016/j.marpolbul.2022.114245**
80. P. G. Ryan, The characteristics and distribution of plastic particles at the sea surface off the Southwestern Cape Province, South Africa. *Mar. Environ. Res.* 25, 249–273 (1988). **https://doi.org/10.1016/0141-1136(88)90015-3**
81. R. J. Morris Plastic debris in the surface waters of the South Atlantic. *Mar. Pollut. Bull.* 185, 114245 (1980) **https://doi.org/10.1016/0025-326X(80)90144-7**
82. M. Eriksen, N. Maximenko, M. Thiel, A. Cummins, G. Lattin, S. Wilson, J. Hafner, A. Zellers, S. Rifman, Plastic pollution in the South Pacific subtropical gyre. *Mar. Pollut. Bull.* 68, 71–76 (2013). **https://doi.org/10.1016/j.marpolbul.2012.12.021**

83. A. Isobe, K. Uchiyama-Matsumoto, K. Uchida, T. Tokai, Microplastics in the Southern Ocean. *Mar. Pollut. Bull.* 114, 623–626 (2017). **https://doi.org/10.1016/j.marpolbul.2016.09.037**

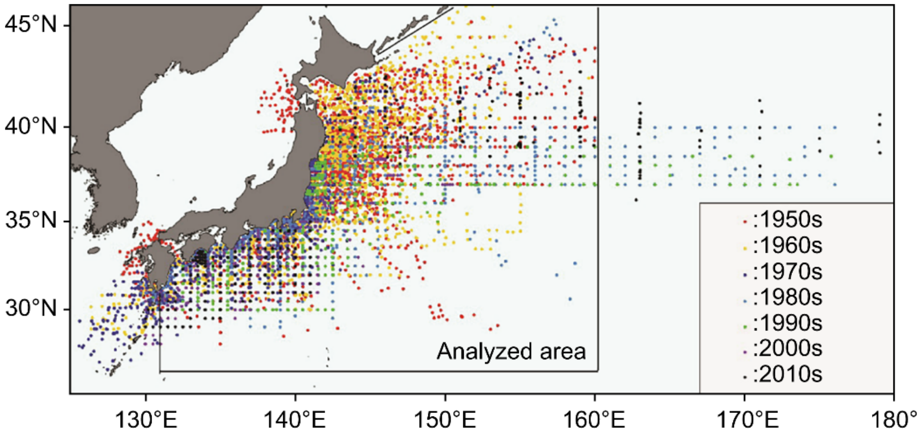

Fig. S1. Location of sampling stations used in this study. Stations that operated in 1949 are included in the group of 1950s. Temporal changes in abundance, size, and morphotypes were analyzed using the samples collected within the study area

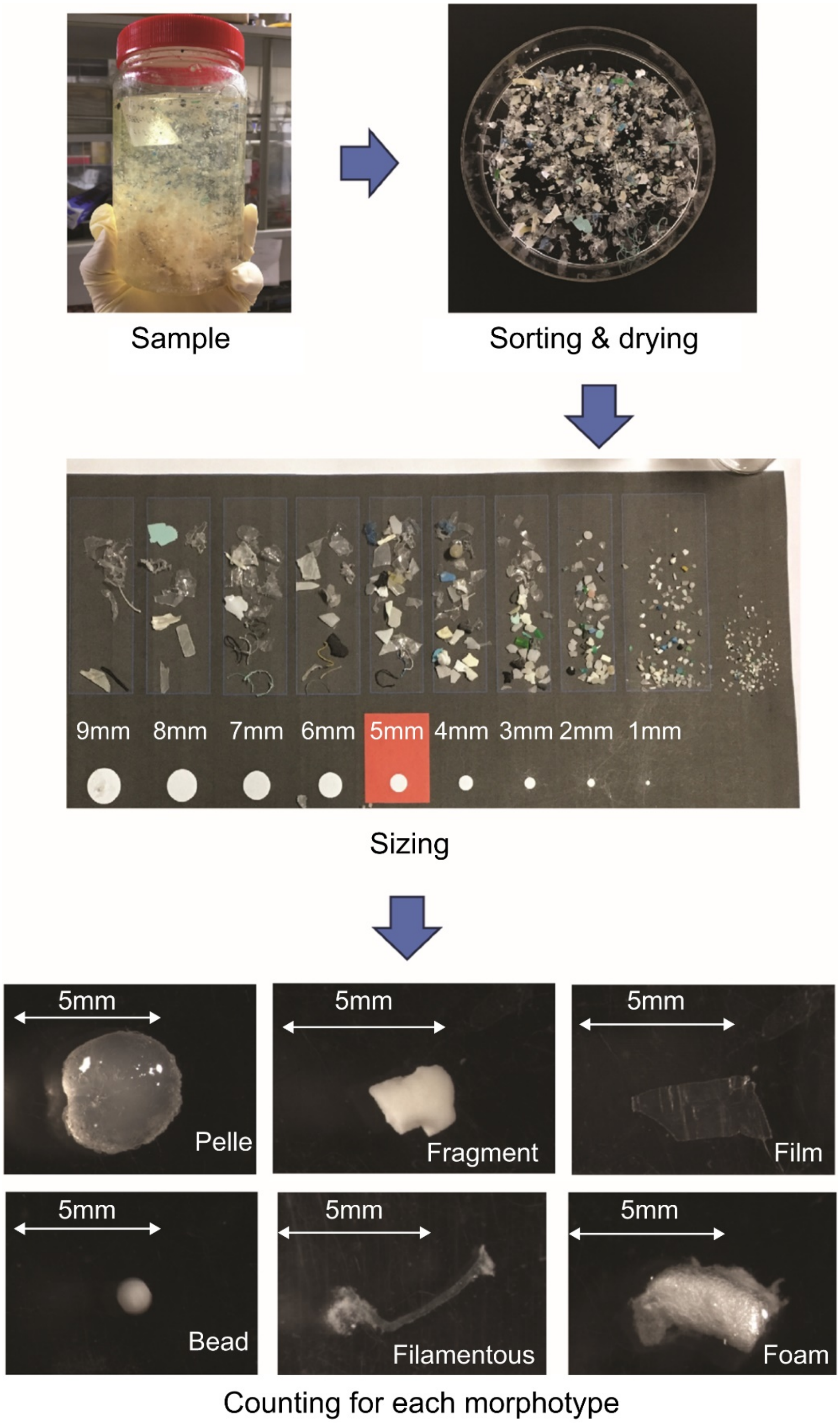

Fig. S2. Procedure for the analysis of plastic debris

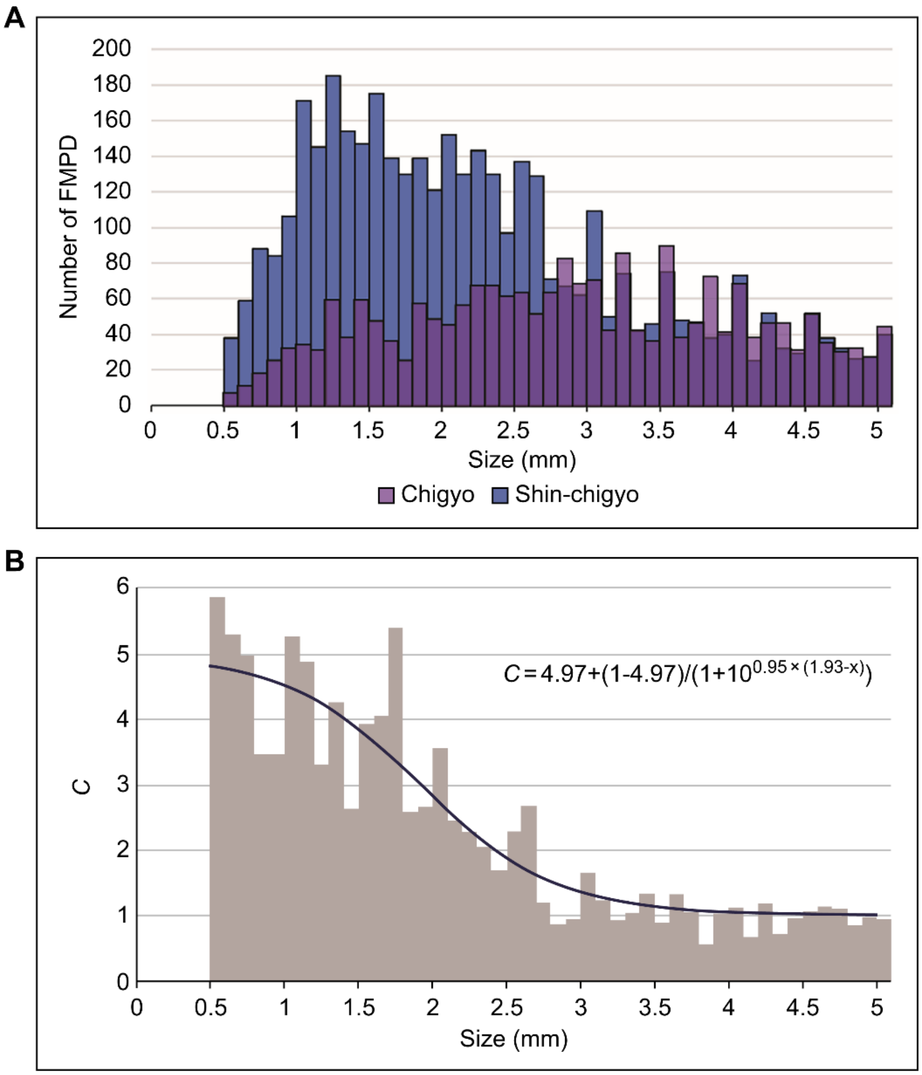

Fig. S3. Data used for inter-calibration of two types of plankton net used during the early phase of monitoring. (A) Comparison of the longest-length distribution of floating plastic debris for the Maruchi (2 mm + 0.33 mm mesh size) and Shin-Chigyo nets (0.45 mm mesh size) collected during 1985–1990. (B) Collection ratios of the two nets for each size-class of floating plastic debris, with the curve fitted for the collection factor (C)

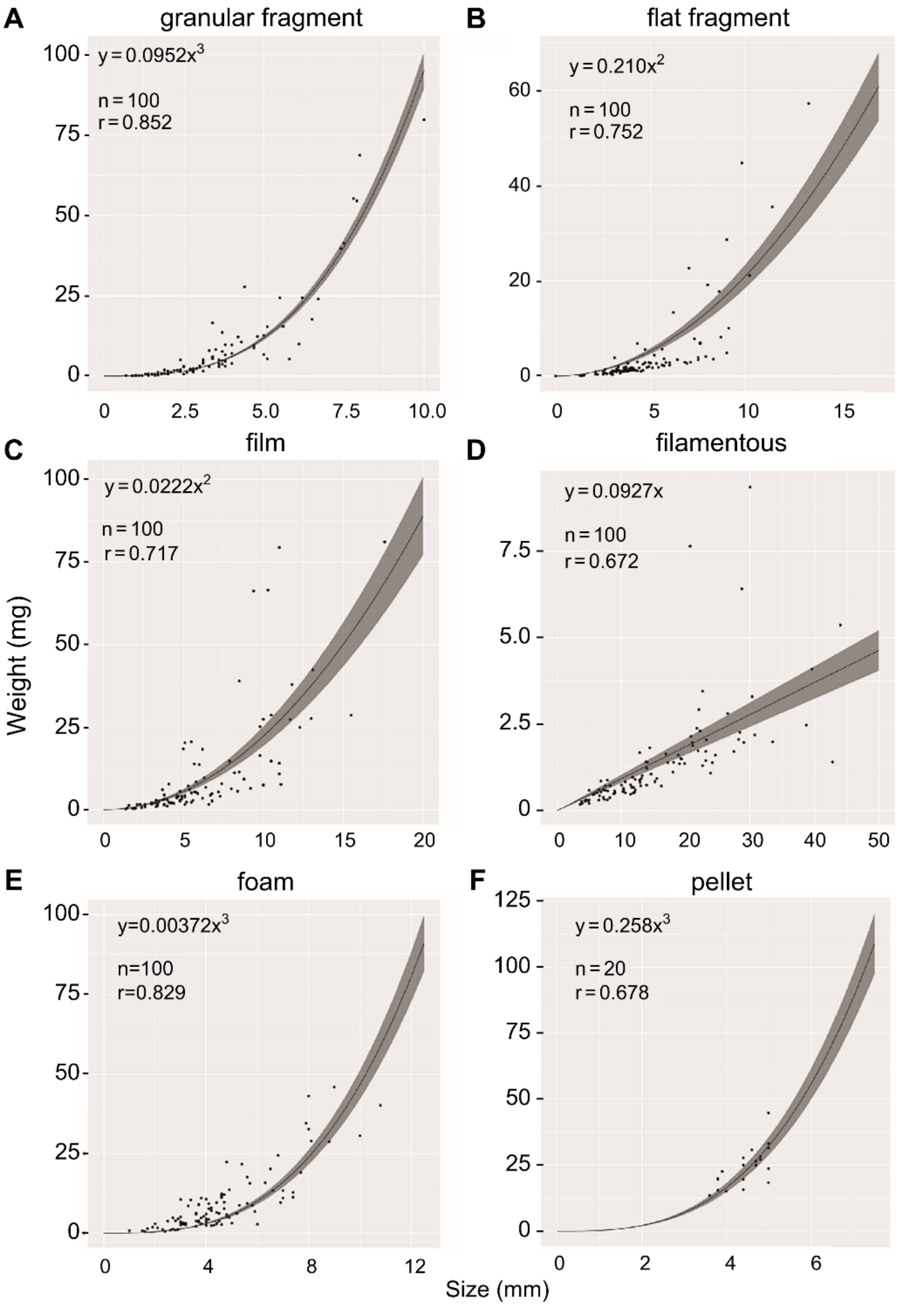

Fig. S4. Length-weight allometric equations used to estimate the plastic concentration (mg/m^2) for Fig. 2C. The equations were established depending on the morphotypes, using samples collected during 1987–2000, roughly corresponding to the middle of the study period. (A) granular fragment, (B) flat fragment, (C) film, (D) filamentous, (E) foam, and (F) pellet; *n* denotes the number of samples, and "r" denotes the correlation coefficient

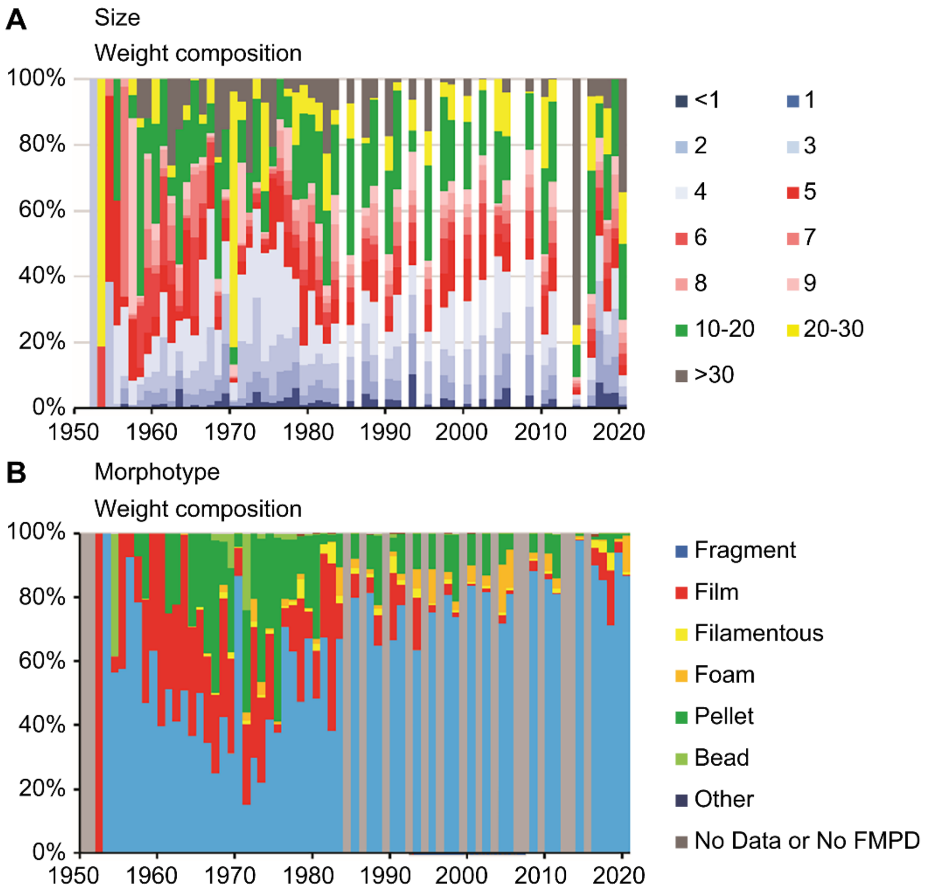

Fig. S5. Temporal change in the weight compositions of the plastic debris collected in the surface waters around Japan in the western North Pacific over the course of 70 years (1949–2020). (A) size class (mm), (B) morphotype of the plastic particles
